# Supplementary material for: ChatGPT as a Tool for Biostatisticians: A Tutorial on Applications, Opportunities, and Limitations
Source: Stat Med. 2025 Oct 23;44(23-24):e70263. doi: 10.1002/sim.70263 (PMC12548020; doi:10.1002/sim.70263)
Supplement: Supplementary file 1 — Data S1. Supporting Information. [file SIM-44-0-s001.zip › Supplementary Materials Additional.zip!/3.1_Systematic_Reviews_and_Meta_aspects/3.1_Data Extraction - Base Prompt o4-mini - Chat 9.pdf]

Use Case Meta-Analysis - Data Extraction - Base Prompt o4-mini - Chat 9

Rudloff et al. - 2009 - Concurrent lobular neoplasia increases the risk of ipsilateral breast cancer recurrence in situ treated with breast-conserving therapy.pdf
PDF

Vidali et al. - 2012 - Conservative treatment of breast ductal carcinoma in situ results of an Italian multi-institutional.pdf
PDF

The aim is to perform a meta-analysis on total ipsilateral local recurrence rates at 10 years in cases of ductal carcinoma in situ

Data inclusion rules are:
To ensure the validity of the follow-up data, the results should be as close to 10 years follow-up (minimum) as the information in each study allows. If there is more than one publication from an institution or group, the most recent study with appropriate follow-up will be used to extract 10-year data.

Please provide:

- the rate of ipsilateral local recurrence for conservative surgery with additional radiotherapy (no other interventions or drugs allowed) after 10 years
- the number of eligible patients
- period of data collection
- duration of follow-up
- type of study
- age of patients (mean or median, maximum and minimum)
- treatment modalities

(Please provide a dataframe with one entry for each study, and column names "study", "rate", "n_patients", "period", "follow_up", "study_type", "age", and "treatment". Do not provide additional text).

```
markdown

| study
| rate                 | n_patients | period                      | follow_up           | study_type
| age                        | treatment
|
|------------------------------------------------------------------------------------------
---------|-------------------|-----------|-------------------------|-------------------|----
-------------------------------------|-------------------------|-----------------------------
---------------------------------------------------------|
```

```
| EBCTCG meta-analysis of four RCTs (NSABP B-17, UKCCCR DCIS, SweDCIS, EORTC 10853)
| 12.9% at 10 years  | 3729       | Trials initiated 1985–1990| 10 years           | Meta-
analysis of randomized trials      | adults (≈25–80 years)    | Breast-conserving surgery +
radiotherapy :contentReference[oaicite:0]{index=0}                                         |
| Vidali et al. 2012
| 10% at 11.3 years  | 586         | Feb 1985–Mar 2000         | median 136 months  | Multi-
institutional retrospective study | median 55 (range 29–84)  | Conservative surgery + whole-
breast RT (median 50 Gy; boost in 50%)  |
```
